# Effects of a Unique Combination of the Whole-Body Low Dose Radiotherapy with Inactivation of Two Immune Checkpoints and/or a Heat Shock Protein on the Transplantable Lung Cancer in Mice

**DOI:** 10.3390/ijms22126309

**Published:** 2021-06-11

**Authors:** Ewa M. Nowosielska, Aneta Cheda, Mateusz Pociegiel, Lukasz Cheda, Paweł Szymański, Antoni Wiedlocha

**Affiliations:** 1 Department of Radiobiology and Radiation Protection, Military Institute of Hygiene and Epidemiology, 4 Kozielska St., 01-163 Warsaw, Poland; aneta.cheda@wihe.pl (A.C.); pawel.szymanski@wihe.pl (P.S.); antoni.wiedlocha@ous-research.no (A.W.); 2 National Centre for Nuclear Research Radioisotope Centre POLATOM, 7A Soltana St., 05-400 Otwock, Poland; mateusz.pociegiel@ncbj.gov.pl; 3 Faculty of Chemistry, Biological and Chemical Research Centre, University of Warsaw, 101 Żwirki i Wigury St., 02-089 Warsaw, Poland; lcheda@chem.uw.edu.pl; 4 Department of Pharmaceutical Chemistry, Drug Analyses and Radiopharmacy, Faculty of Pharmacy, Medical University of Lodz, 1 Muszyńskiego St., 90-151 Lodz, Poland; 5 Department of Molecular Cell Biology, Institute for Cancer Research, The Norwegian Radium Hospital, Oslo University Hospital, Montebello, 0379 Oslo, Norway; 6 Centre for Cancer Reprograming, Institute of Clinical Medicine, Faculty of Medicine, University of Oslo, Montebello, 0379 Oslo, Norway

**Keywords:** low dose irradiation, immune checkpoints, anti HSP90 chaperone, lung tumor, mice

## Abstract

Non-small cell lung cancer (NSCLC) continues to be the leading cause of cancer death worldwide. Recently, targeting molecules whose functions are associated with tumorigenesis has become a game changing adjunct to standard anti-cancer therapy. As evidenced by the results of preclinical and clinical investigations, whole-body irradiations (WBI) with X-rays at less than 0.1–0.2 Gy per fraction can induce remissions of various neoplasms without inciting adverse side effects of conventional chemo- and radiotherapy. In the present study, a murine model of human NSCLC was employed to evaluate for the first time the anti-neoplastic efficacy of WBI combined with inactivation of CTLA-4, PD-1, and/or HSP90. The results indicate that WBI alone and in conjunction with the inhibition of the function of the cytotoxic T-lymphocyte antigen-4 (CTLA-4) and the programmed death-1 (PD-1) receptor immune checkpoints (ICs) and/or heat shock protein 90 (HSP90) markedly reduced tumorigenesis in mice implanted by three different routes with the syngeneic Lewis lung cancer cells and suppressed clonogenic potential of Lewis lung carcinoma (LLC1) cells in vitro. These results were associated with the relevant changes in the profile of pro- and anti-neoplastic immune cells recruited to the growing tumors and the circulating anti- and pro-inflammatory cytokines. In contrast, inhibition of the tested molecular targets used either separately or in combination with each other did not exert notable anti-neoplastic effects. Moreover, no significant synergistic effects were detected when the inhibitors were applied concurrently with WBI. The obtained results supplemented with further mechanistic explanations provided by future investigations will help design the effective strategies of treatment of lung and other cancers based on inactivation of the immune checkpoint and/or heat shock molecules combined with low-dose radiotherapy.

## 1. Introduction

Lung cancer is the leading cause of cancer mortality in men aged ≥ 40 years and women aged ≥60 years, causing far more deaths than other dominant cancers of men and women [1]. The great majority (80–85%) of newly diagnosed cases of lung cancer are non-small cell carcinomas (NSCLC) [2]. Currently, patients with NSCLC are treated with all the three conventional therapeutic methods, i.e., surgery, chemotherapy, and radiotherapy. In the latter case, localized irradiations of the tumor with large doses of X- or gamma rays, which are divided into smaller fractions applied over several days or weeks, are used [3]. Such irradiations, often combined with chemotherapy, can lead to more or less durable remissions [4,5]. Generally, however, the prognosis for NSCLC patients, which are often diagnosed at an advanced stage of the disease, remains poor [2,6].

In contrast to local irradiations of the tumor with doses in the range of 1–2 Gy per fraction, exposures of the whole body of patients at low doses, i.e., not exceeding 0.1–0.2 Gy per fraction [7], do not kill or injure normal cells and are devoid of such adverse effects of the high-dose irradiations as inflammation, immunosuppression, and secondary cancers (reviewed in [8,9]). Indeed, as evidenced by the results of numerous animal studies including our own (reviewed in [8]) and, especially, of more than forty reliable clinical trials performed since the early 1930s having documented that fractionated whole-body irradiations (WBI) improve long-term overall survival and cure rates in patients with advanced hematological and solid neoplasms (reviewed in [9]). Arguably, a crucial underlying mechanism of such effects is up-regulation of the function of the immune system, the organism’s most important guardian against cancer (reviewed in [8,10,11]).

However, the WBI-induced immunostimulation may also possibly trigger the activity of immune checkpoints (ICs), which dampen T cell activation and thereby allow tumors to escape the elimination by cytotoxic lymphocytes. Consequently, in the last decade, inhibition of the function of ICs has become an important novel form of therapy of patients with malignancies, including lung cancer (reviewed in [12,13,14,15,16,17,18,19]). As demonstrated by the accumulated evidence, application of monoclonal antibodies against the two best characterized ICs, the cytotoxic T-lymphocyte antigen-4 (CTLA-4) and the programmed death-1 (PD-1) receptor, constitutes a major breakthrough in experimental and clinical oncology and demonstrates the potential of immune-mediated therapies in achieving durable cancer remissions [13,16,20,21,22,23]. Along these advances, effects of the inhibition of ICs combined with radiotherapy have been investigated in both pre-clinical and clinical settings. The results demonstrate that the immune checkpoint blockade in conjunction with localized irradiations of a bulk tumor mass is more efficient than standard chemo- and/or radiotherapy [18,22,24,25,26,27,28,29,30]. Thus far, however, no experimental or clinical studies have been designed to evaluate the effects of the combined application of WBI and anti-immune checkpoint(s) molecules.

In addition to stimulation of the anti-neoplastic immunity, blockade of intracellular molecules that promote cancer progression has been tested in preclinical models as a potential anti-cancer treatment. Among the molecular targets of such treatment are heat shock proteins (HSPs), such as HSP90, which facilitate the function of oncoproteins (reviewed in [31,32,33]) and have been considered as co-factors for the development and progression of the malignant phenotype [24,34,35]. Indeed, poor prognosis in patients with multiple tumors, including lung cancer, has been coupled to overexpression of HSP90 [36,37]. Consequently, the results of both preclinical and clinical studies have demonstrated that inhibition of the function of HSP90 can stifle progression of lung carcinoma and other malignancies [33,38,39,40,41,42].

Hence, the aim of the present investigation was to evaluate the therapeutic efficacy of WBI of the lung tumor-bearing mice combined with the blockade of the function of one or two ICs and/or the HSP90 chaperone. To this end, we employed syngeneic Lewis lung carcinoma (LLC1) cells injected into C57BL/6 mice via three different routes: (a) intravenously (i.v.), to assess the development of the induced tumor colonies in the lungs, spleen, and liver; (b) orthotopically (o.t.), to evaluate the growth of a primary tumor in the injected lung and its possible metastases in other organs; and (c) subcutaneously (s.c.), to follow the development of a measurable primary tumor and the occurrence of its possible metastases to internal organs. Clonogenic potential in vitro of the tumor cells obtained from the mice injected with the LLC1 cells was also estimated. In addition, given the importance of immune cells in the tumor microenvironment (reviewed in [43,44,45]), the tumor-infiltrating T lymphocytes (TILs) and natural killer (NK) lymphocytes as well as tumor-associated macrophages (TAMs) were quantified in the pulmonary and subcutaneously growing tumors in mice injected with LLC1 cells. Additionally, the levels of the selected pro- and anti-inflammatory cytokines involved in immune surveillance were estimated.

## 2. Results

**Figure 1:**
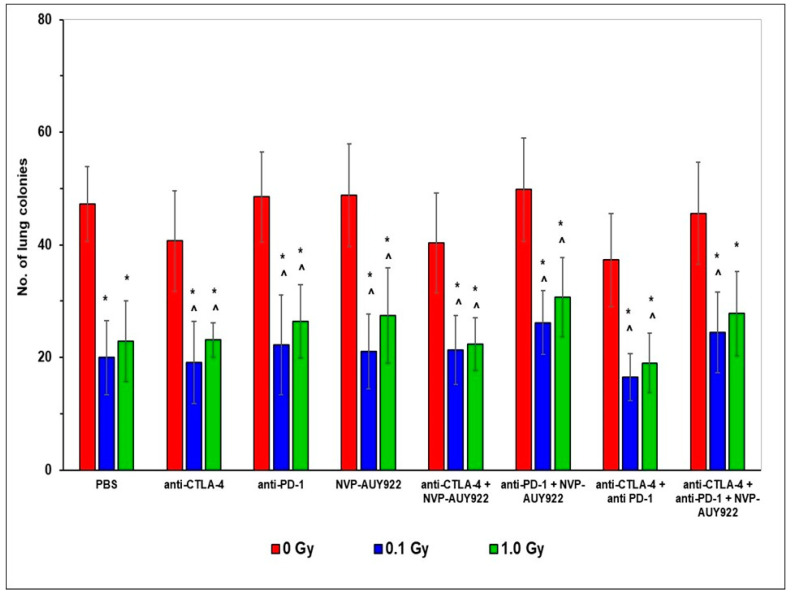
Tumor colonies in the lungs of C57BL/6 mice intravenously (i.v.) injected with Lewis lung carcinoma (LLC1) cells and treated with blockers of the immune checkpoints and/or heat shock protein 90 (HSP90) combined with whole-body irradiations (WBI) at total doses of 0.1 and 1.0 Gy. Mean values ± SD obtained from experiments conducted on ten mice per group are presented. * indicates statistically significant (*p* < 0.05) difference from the results obtained in the sham-exposed mice injected with PBS; ^ indicates statistically significant (*p* < 0.05) difference from the results obtained in the sham-exposed mice injected with the same inhibitory molecules.

As shown in Figure 1, fractionated WBI at total doses of 0.1 and 1.0 Gy led to the significant reduction in the number of tumor colonies developing in the lungs of the C57BL/6 mice after the i.v. injection of the LLC1 cells. In contrast, neither of the inhibitors of the function of CTLA-4, PD-1, or HSP90 applied separately or in combination with each other appeared to significantly suppress the development of the tumor colonies. Moreover, application of these inhibitors together with WBI did not significantly affect the anti-neoplastic effect of the latter, although the number of tumors in the lungs tended to be lower in mice irradiated at total doses of 0.1 or 1.0 Gy and concurrently treated with the anti-CTLA-4 and anti-PD-1 molecules. Noticeably, the use of WBI with any of the tested blockers, applied alone or in combination, was significantly more efficient in suppressing the development of tumor colonies than either of the inhibitors used alone. This effect tended to be more pronounced when the animals were irradiated at the smaller total dose of 0.1 Gy (Figure 1).

In this set of experiments, no macroscopic tumor metastases were observed in the spleens and livers of mice i.v. injected with the LLC1 cells.

As demonstrated by the data presented in Table 1, the in vitro clonogenic capacity (CC) of the tumor cells obtained from the lungs of mice i.v. injected with the LLC1 cells was markedly suppressed by WBI at both total doses. Compared to the effects of the inhibitors of the function of CTLA-4, PD-1, and/or HSP90 used alone or in combination with each other, additional treatment with WBI appeared to be clearly more efficient in suppressing the CC of the lung-derived tumor cells. This latter effect was apparently more pronounced when the mice were irradiated at 0.1 Gy rather than 1.0 Gy of the total dose (Table 1).

In this set of experiments, no tumor cell colonies (clones) developed in vitro when the plates were seeded with cell suspensions obtained from the spleens or livers of the LLC1 cells-injected mice from all the experimental and control groups.

**Figure 2:**
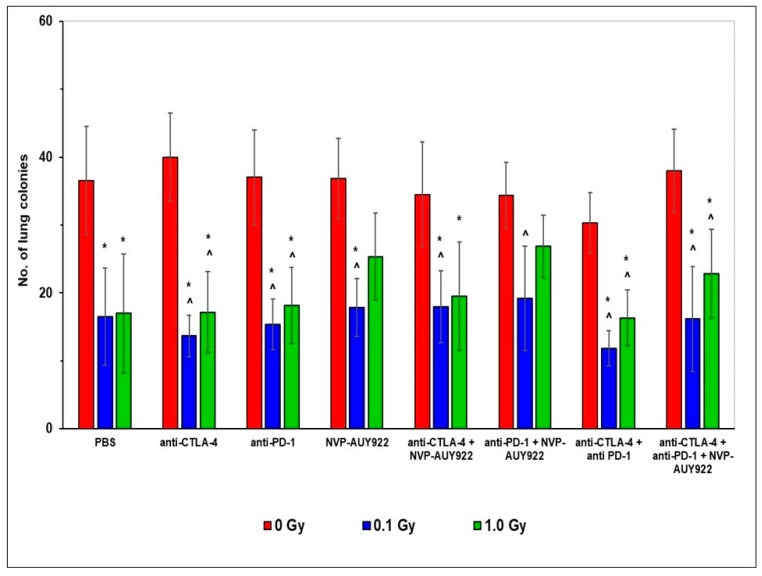
Tumor colonies in the lungs of C57BL/6 mice orthotopically (o.t.) injected with LLC1 cells and treated with blockers of the immune checkpoints and/or HSP90 combined with WBI at total doses of 0.1 and 1.0 Gy. Mean values ± SD obtained from experiments conducted on ten mice per group are presented. * indicates statistically significant (*p* < 0.05) difference from the results obtained in the sham-exposed mice injected with PBS; ^ indicates statistically significant (*p* < 0.05) difference from the results obtained in the sham-exposed mice injected with the same molecule or combination of the molecules.

After o.t. injection of the LLC1 cells, not only the primary tumor, but also metastatic colonies were expected to develop in the lungs, liver, and spleen. Hence, total numbers of macroscopic tumor hotbeds were counted in these organs. As shown in Figure 2, similar to the results presented in Figure 1, fractionated WBI at total doses of 0.1 and 1.0 Gy led to the significant reduction in the number of tumors developing in the lungs of the C57BL/6 mice o.t. injected with the LLC1 cells. In contrast, neither of the inhibitors of CTLA-4, PD-1, and/or HSP90 applied solely or in combination with each other appeared to suppress the development of the lung tumors. Moreover, addition of the inhibitors to WBI did not appear to synergistically impact the tumoricidal effect of the latter, although exposure at the smaller total dose of WBI along with application of the anti-CTLA-4 molecules, especially when combined with the anti-PD-1 but not with the anti-HSP90 molecules, was clearly more efficient in this regard. Notably, the anti-neoplastic effect of WBI used together with any of the tested blockers, applied both alone and in combination, was significantly more pronounced than the effect of the inhibitors used separately or with one another. Again, combination with WBI at the smaller total dose tended to be more efficient in this regard (Figure 2).

As in the case of the standard lung colony assay, no macroscopic tumor metastases were detected in the spleens and livers of mice orthotopically injected with LLC1 cells.

Similar to the results shown in Table 1, CC in vitro of the tumor cells obtained from the lungs directly implanted with the LLC1 cells was suppressed by WBI at both total doses, although the effect was significant only after exposure at 0.1 Gy total dose (Table 2). In contrast, in the majority of cases, sole application of any of the anti-CTLA-4, anti-PD-1, and/or anti-HSP90 molecules used either alone or in combination with each other did not affect the clonogenic potential of the lung-derived tumor cells. The only exception was the combined application of the anti-CTLA-4 and the anti-PD-1 molecules which significantly thwarted the growth of the tumor clones in vitro. Compared to the effects of the molecular inhibitors used alone, additional treatment with WBI appeared to be clearly more efficient in suppressing the CC of the lung-derived tumor cells. This latter effect was noticeably more pronounced when mice were irradiated at 0.1 Gy rather than 1.0 Gy of the total dose (Table 2). Moreover, combined blockade of the function of CTLA-4 and PD-1 applied concurrently with WBI resulted in the synergistic reduction of the clonogenic potential of the lung-derived tumor cells, the effect being more pronounced in mice exposed at 0.1 Gy total dose.

As in the case of the standard lung colony assay, in all the experimental and control groups, no tumor cell colonies (clones) developed in vitro when the plates were seeded with cell suspensions obtained from the spleens or livers of mice whose left lung was o.t. injected with the LLC1 cells.

**Figure 3:**
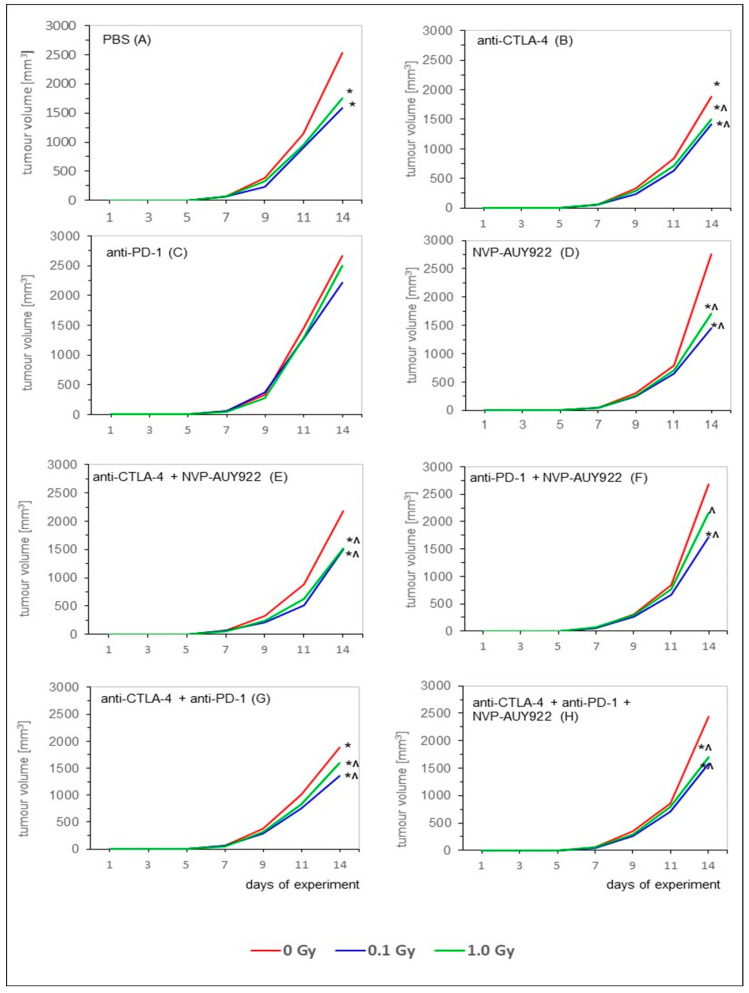
Tumor growth in C57BL/6 mice s.c. injected with LLC1 cells and exposed to WBI at total doses of 0.1 or 1.0 Gy combined with the blockade of the function of one or two immune checkpoints and/or HSP90. The curves formed from mean values of the tumor volumes measured in 6 mice per group are presented. * indicates statistically significant (*p* < 0.05) difference from the results obtained in the sham-exposed mice injected with PBS; ^ indicates statistically significant (*p* < 0.05) difference from the results obtained in the sham-exposed mice injected with the same molecule or combination of the molecules.

Finally, as indicated in Figure 3, WBI at both 0.1 and 1.0 Gy total doses administered in five daily fractions exhibited the significant anti-neoplastic activity as judged by the reduced volume of the s.c. growing tumors developing from the implanted LLC1 cells (Figure 3A). Comparable effect was detected after inhibition of the function of CTLA-4 used alone and in combination with the blockade of the PD-1 receptor or HSP90 protein. Noticeably, in most of the cases (the only exception being combination with the anti-PD-1 molecules), concurrent use of WBI at both total doses and the inhibitors exerted the markedly more pronounced anti-neoplastic effect than either combination of the inhibitory molecules used alone. Once again, combination of these molecules with WBI at the smaller total dose tended to be more efficient in this regard (Figure 3).

No macroscopic tumor colonies in the lungs, spleens, and livers nor the clones of the LLC1 cells of mice, which had been subcutaneously implanted with the LLC1 cells, were detected.

As shown in Table 3, the CD4+/CD8+ T lymphocytes ratio among the cells obtained from the lungs of mice i.v. or o.t. injected with the LLC1 cells was markedly decreased after WBI at both total doses. The effect was also significant when irradiation was accompanied by application of anti-CTLA-4 alone or in combination with anti-PD-1 or anti-HSP90 molecules. In contrast, the sole application of any of the inhibitory molecules used either alone or in combination with each other did not affect the CD4+/CD8+ ratios. Similarly, the CD4+/CD8+ ratios determined among the cells obtained from the subcutaneously implanted tumors were not affected by the treatment with any of the anti-CTLA-4, anti-PD-1, and/or anti-HSP90 molecules used either alone or in combination with each other. The only exception was the combined use of the anti-CTLA-4 and the anti-PD-1 molecules which significantly decreased the CD4+/CD8+ ratio which was further significantly reduced by WBI at 1 Gy total dose. The latter reduction was also noted when WBIs accompanied the application of any of the anti-CTLA-4, anti-PD-1, and/or anti-HSP90 molecules used either alone or in combination with each other, but the CD4+/CD8+ ratios were still somewhat higher than the one detected after the sole irradiation at 1.0 total dose.

**Figure 4:**
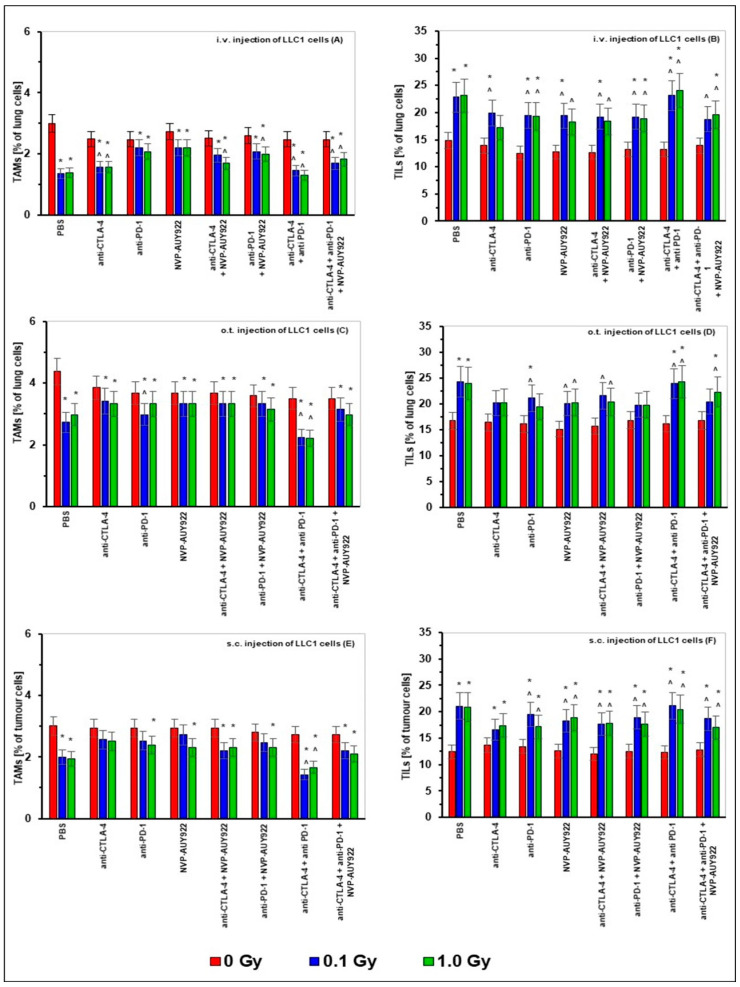
The percentages of tumour- associated macrophages (TAMs) (panels **A**,**C**,**E**) and tumour-infiltrating lymphocytes (TILs) (panels **B**,**D**,**F**) in the lungs after i.v. (panels **A**,**B**) or o.t. (panels **C**,**D**) injection with LLC1 cells or in tumors after s.c. (panels **E**,**F**) injection of LLC1 cells to C57BL/6 mice treated with blockers of the immune checkpoints and/or HSP90 combined with WBI at total doses of 0.1 and 1.0 Gy. The bars indicate mean percentages of TAMs and TILs calculated for six (i.v. and o.t. injection of tumor cells) or three (s.c. injection of tumor cells) mice per group are presented. * indicates statistically significant (*p* < 0.05) difference from the results obtained in the sham-exposed mice injected with PBS; ^ indicates statistically significant (*p* < 0.05) difference from the results obtained in the sham-exposed mice injected with the same inhibitory molecule or a combination of these molecules.

As indicated in Figure 4, the numbers of TAMs (CD11b+Ly6C+Ly6G+) in the lungs of mice i.v. or o.t. injected with LLC1 cells as well as in s.c. implanted tumors were significantly reduced by WBI at both total doses of X-rays, whereas sole application of any of the anti-CTLA-4, anti-PD-1, and/or anti-HSP90 molecules used either alone or in combination with each other did not affect these numbers. WBI combined with application of any of the anti-CTLA-4, anti-PD-1, and/or anti-HSP90 molecules used alone or in combination, resulted in the majority of cases in the increased percentages of TAMs in both the pulmonary and subcutaneous tumors, the effect being most pronounced when irradiations at both total doses were combined with application of anti-CTLA-4 and anti-PD-1 molecules. In turn, the numbers of TILs (CD3+CD45+) in the lungs of mice i.v. or o.t. injected with LLC1 cells as well as in s.c. implanted tumors were significantly elevated by WBI at both total doses of X-rays. In contrast, sole application of any of the anti-CTLA-4, anti-PD-1 and/or anti-HSP90 molecules used either alone or in combination with each other did not affect these cells’ numbers. However, irradiations combined with application of any of the anti-CTLA-4, anti-PD-1, and/or anti-HSP90 molecules used alone or, in most cases, in combination with each other, increased the percentages of TILs in both the lungs and subcutaneous tumors.

**Figure 5:**
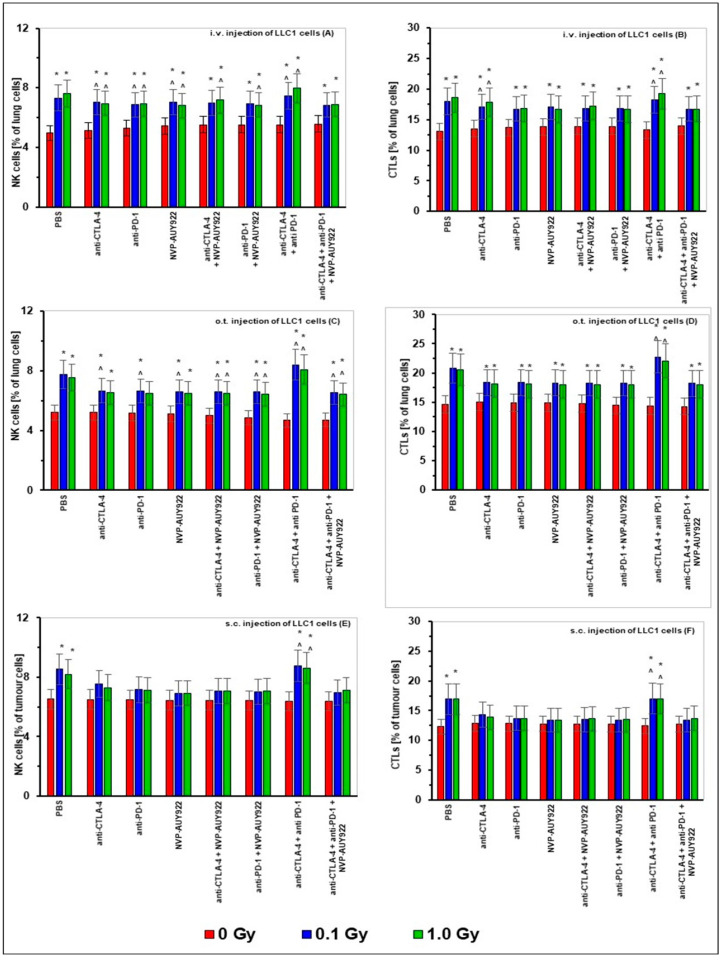
The percentages of natural killer (NK) cells (panels **A**,**C**,**E**) and cytotoxic T lymphocytes (CTLs) (panels **B**,**D**,**F**) in the lungs after i.v. (panels **A**,**B**) or o.t. (panels **C**,**D**) injection with LLC1 cells or in tumors after s.c. (panels **E**,**F**) injection of LLC1 cells to C57BL/6 mice treated with blockers of the immune checkpoints and/or HSP90 combined with WBI at total doses of 0.1 and 1.0 Gy. The bars indicate mean percentages of NK cells and CTLs calculated for six (i.v. and o.t. injection of tumor cells) or three (s.c. injection of tumor cells) mice per group are presented. * indicates statistically significant (*p* < 0.05) difference from the results obtained in the sham-exposed mice injected with PBS; ^ indicates statistically significant (*p* < 0.05) difference from the results obtained in the sham-exposed mice injected with the same inhibitory molecule or a combination of these molecules.

As in the case of TILs, percentages of NK (CD49b+CD335+) and CTL (CD8+Cd178+) lymphocytes in the lungs of mice i.v. or o.t. injected with LLC1 cells were significantly elevated by WBI at both total doses of X-rays (Figure 5A–D). In contrast, sole application of any of the anti-CTLA-4, anti-PD-1, and/or anti-HSP90 molecules used either alone or in combination with each other did not affect the numbers of these cells. However, irradiations combined with application of any of the inhibitors used alone and in combination with each other increased, in the majority of cases, the relative numbers of NK cells and CTLs in the lungs. The latter effect seemed to be most pronounced when WBIs at both total doses were accompanied by application of anti-CTLA-4 in combination with anti-PD-1 molecules. Likewise, the numbers of NK cells and CTLs in the s.c. implanted tumors were significantly elevated by WBI at both total doses of X-rays. An even more pronounced effect was detected when irradiations were accompanied by the combined application of anti-CTLA-4 and anti-PD-1 molecules. In contrast, in the majority of cases, the sole application of any of the anti-CTLA-4, anti-PD-1, and/or anti-HSP90 molecules used either alone or in combination with each other did not significantly affect the numbers of NK cells and CTLs in the subcutaneously growing tumors (Figure 5E,F).

**Figure 6:**
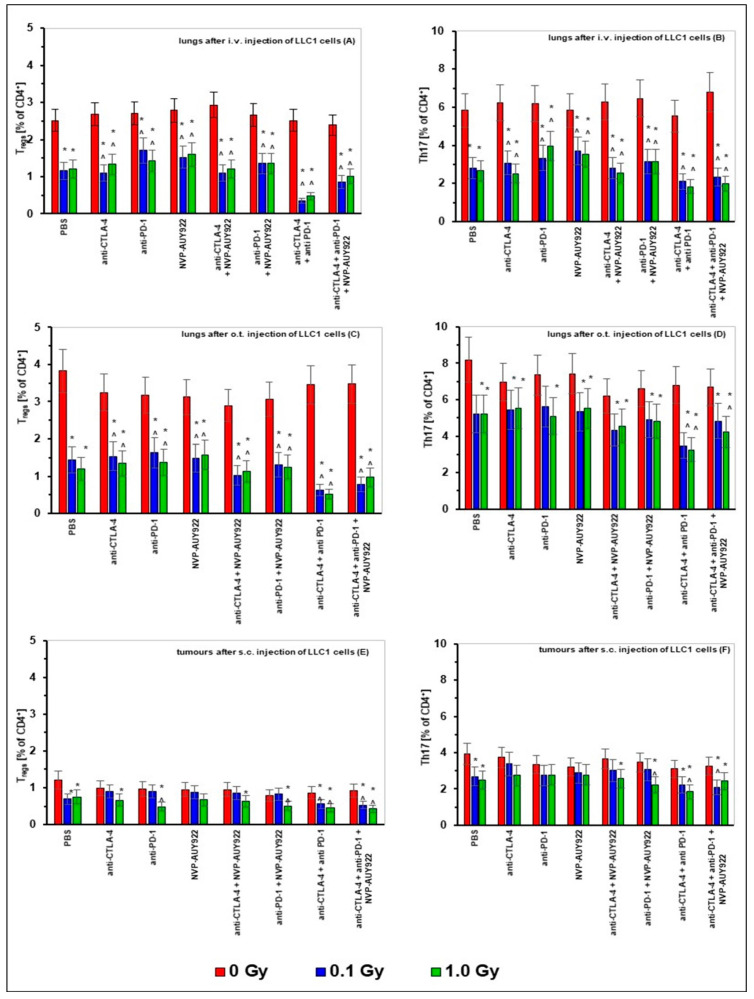
The percentages of regulatory T lymphocytes (Tregs) (panels **A**,**C**,**E**) and helper T lymphocytes (Th17 cells) (panels **B**,**D**,**F**) in the lungs after i.v. (panels **A**,**B**) or o.t. (panels **C**,**D**) injection with LLC1 cells or in tumors after s.c. (panels **E**,**F**) injection of LLC1 cells to C57BL/6 mice treated with blockers of the immune checkpoints and/or HSP90 combined with WBI at total doses of 0.1 and 1.0 Gy. The bars formed from mean values of the Tregs and Th17 cells measured in 6 (i.v. and o.t. injection of tumor cells) or 3 (s.c. injection of tumor cells) mice per group are presented. * indicates statistically significant (*p* < 0.05) difference from the results obtained in the sham-exposed mice injected with PBS; ^ indicates statistically significant (*p* < 0.05) difference from the results obtained in the sham-exposed mice injected with the same molecule or combination of the molecules.

As shown in Figure 6, the population of Treg CD4+CD25+FoxP3+) and Th17 (CD4+IL17A+) lymphocytes in the lungs of mice i.v. or o.t. injected with LLC1 cells as well as in s.c. implanted tumors were significantly reduced by WBI at both total doses of X-rays. This reduction did not seem to be significantly affected by the concurrent application of the inhibitory molecules used either alone or in combination with the exception of the effect of the combined use of the anti-CTLA-4 and anti-PD-s molecules on the numbers of Treg cells. Notably, sole application of the anti-CTLA-4, anti-PD-1, and/or anti-HSP90 molecules used either alone or in combination with each other did not markedly impact on the numbers of Treg and T17 lymphocytes recovered from the tumorous tissues (Figure 6A–F).

Serum levels of the putatively anti-inflammatory TGF-β (Table S1) and IL-10 (Table S2) in mice i.v., o.t., or s.c. injected with LLC1 cells were significantly reduced by WBI at 0.1 and 1.0 Gy total doses of X-rays, the effect being more pronounced after irradiation with the former total dose. In contrast. In contrast, the sole application of any of the inhibitors used either alone or in combination with each other did not affect these cytokines’ levels. However, WBI combined with application of any of the anti-CTLA-4, anti-PD-1, and/or anti-HSP90 molecules used alone or in combination with each other decreased the secretion of these cytokines, the effect being most pronounced when irradiations at both total doses were combined with concurrent application of anti-CTLA-4 and anti-PD-1 molecules.

In turn, the levels of the pro-inflammatory TNF-α (Table S3) and IFN-γ (Table S4) in the serum collected from mice i.v., o.t., or s.c. injected with LLC1 cells were significantly elevated by WBI at both total doses of X-rays, whereas sole application of any of the inhibitory molecules used either alone or in combination with each other did not affect the levels of these cytokines. However, WBI combined with application of any of the anti-CTLA-4, anti-PD-1, and/or anti-HSP90 molecules used alone or in combination with each other, in the majority of cases, led to the increased production of these cytokines; the effect being most pronounced when irradiations at both total doses were combined with concurrent application of anti-CTLA-4 and anti-PD-1 molecules.

No differences between the examined groups were detected in the serum levels of the pro-inflammatory IL-6 (Table S5) in mice i.v., o.t., or s.c. injected with LLC1 cells, exposed to WBI, and treated with anti-CTLA-4, anti-PD-1, and/or anti-HSP90 molecules used either alone or in combination with each other.

In this set of experiments, the serum levels of Il-2, IL-4, and IL-17A were assessed below the detection limit of the method in all the examined groups.

## 3. Discussion

The present investigation was undertaken in order to assess for the first time the presumable anti-neoplastic effects of the combination of the low-dose whole-body irradiation (WBI) with X-rays combined with the blockade of the function of one or two immune checkpoints and/or a heat shock protein. Our studies were performed using a reliable preclinical model of the human non-small cell lung cancer [46]. In this model, Lewis lung cancer (LLC1) cells derived from an epidermoid carcinoma that had spontaneously developed in the lung of a C57BL mouse were injected through three different routes into syngeneic C57BL/c mice. Between seven and eleven days later, i.e., when the subcutaneously growing tumors were palpable, the mice were exposed to WBI and injected with the inhibitors of the ICs or HSP90. Progression of the tumors developing in the lungs and other internal organs or under the skin of the animals as well the clonogenic potential of these cells in vitro were estimated. Additionally, various immune cells infiltrating the tumors as well as serum levels of the selected pro- and anti-inflammatory cytokines were quantified.

Our first important observation was that five subsequent WBIs of the relatively radioresistant C57BL/6 mice [47,48] at daily doses of 0.02 and 0.2 Gy of X-rays (i.e., at 0.1 and 1.0 Gy total doses) significantly suppressed the in vivo growth of syngeneic LLC1 cells and, the effect being detectable in mice whose tumors were induced either by i.v., o.t., or s.c. injection of the LLC1 cells. Likewise, the clonogenic capacity in vitro of the tumor cells obtained from the lungs of mice injected with LLC1 cells was markedly inhibited by the WBIs. Notably, the reduced tumorigenesis in vivo was more pronounced after irradiations at the lower compared to the higher total dose of X-rays, the effect being paralleled by the lower clonogenic potential in vitro of LLC1 cells obtained from the lungs of mice irradiated at 0.02 Gy per day compared to the potential of the cells recovered from mice exposed at 0.2 Gy per day (Figure 1, Figure 2 and Figure 3, Table 1 and Table 2). Apparently, some in vivo factors were induced by the lower-dose irradiations, which more effectively than the higher-dose exposures, suppressed the potential of the tumor cells to grow in vitro and in vivo. This notion is supported by multiple observations that exposures to low-LET radiation at ≤0.1 Gy per fraction more potently suppresses cancer growth in experimental animals and human patients than do irradiations at higher daily doses (reviewed in [8,9]) Generally, these results corroborate the findings from our earlier experiments conducted in both C57BL/6 and the more radiosensitive BALB/c mice exposed to both single and multiple WBIs with X-rays at 0.1 and 0.2 Gy and assayed for the development of the induced neoplastic colonies in the lungs [49,50,51,52]. In fact, the present results expand our previous observations in that the anti-tumor effects were expressed not only after i.v., but also after o.t. and s.c. injection of LLC1 cells. In the previous investigations, the mice were irradiated before inoculation of the syngeneic tumor cells and, as indicated by the up-regulated activities of NK lymphocytes and cytotoxic macrophages obtained from the spleen and peritoneal cavities, respectively, of the mice, it was the systemic stimulation of innate immunity that was associated with the anti-neoplastic effect of the WBIs. In contrast, in the present study, C57BL/6 mice were exposed to X-rays several days after injection of the tumor cells and still the anti-neoplastic efficacy of WBIs could be clearly demonstrated in mice in which the tumors were induced by three different routes of implantation of LLC1 cells. This effect was accompanied by the significant down- and up-regulation of the numbers of the TAMs and TILs, respectively, in both the pulmonary and subcutaneous tumors. Notably, after WBIs, the relative numbers of Tregs and Th17 cells as well as the CD4+/CD8+ ratio among the recovered TILs were down-regulated, whereas the numbers of the CTLs and NK lymphocytes were elevated. Furthermore, after WBIs at both total doses, decreased levels of IL-10 and TGF-β and elevated levels of IFN-γ and TNF-α were detected in the sera of the tested mice. These results suggest that WBIs employed in the present study stimulated recruitment to the growing tumors of cytotoxic lymphocytes while down-regulating the influx of cells that might promote inflammation and tumor growth. At the same time, the irradiations prompted the secretion to the circulation of two pro-inflammatory cytokines and suppressed the secretion of two anti-inflammatory cytokines. The potential impact of the modified levels of the systemic cytokines on the growth of the i.v., o.t., and/or s.c. injected LLC1 cells needs to be clarified in future studies.

The second important finding of the present investigation is that application of any one or any combination of the tested anti-immune checkpoint and anti-HSP90 molecules unassisted by a parallel WBI did not generally impact the growth of LLC1 cells in the lungs of the i.v. or o.t. injected mice. Noticeably, injections of a single inhibitory agent, especially the anti-HSP molecule NVP-AU922, were the least effective. However, concurrent application of the anti-CTLA-4 and anti-PD-1 molecules appeared to markedly inhibit the formation of the in vitro colonies by the tumor cells obtained from the lungs of the i.v.- or o.t.-injected mice (Table 1 and Table 2). Moreover, such a combined blockade of the two immune checkpoints significantly inhibited the development of subcutaneously growing tumors. (Figure 3G). Although the growth of these tumors was also thwarted by a sole application of the anti-CTLA-4 molecule (Figure 3B), the effect was not accompanied by suppression of the above described clonogenic capacity of LLC1 cells in vitro. The finding of the more pronounced anti-neoplastic efficiency of the combined inactivation of two complementary checkpoints is in accord with the observations that such a simultaneous blockade shows synergistic effects [53,54]. Noticeably however, in contrast to the effects of WBIs used as the only treatment, sole application of any one or any combination of the tested inhibitory molecules did not seem to produce significant changes in the numbers of the tumor infiltrating immune cells or the serum levels of the tested cytokines. The general lack of the tumor-inhibitory activity of the blockers of the CTLA-4, PD-1, and HSP90 molecules in mice i.v. and o.t. injected with LLC1 cells is surprising in view of the results of studies indicating that single and especially combined applications of such blockers were able to successfully enhance the response rates and survival of patients with various malignancies including lung cancer (reviewed in [13,15,16,17,33,41]). One possible explanation of this discrepancy is that the CTLA-4- and/or PD-1-related signaling pathways are not activated in tumors developing in mice implanted by certain routes with the LLC1 or, as was demonstrated for HSP90, blockade of the function of such molecules does not affect the growth of such cells in mice [55]. This possibility might also explain the general observation from the clinical trials that therapy targeted at immune checkpoints has been effective only in a fraction of the lung cancer patients and that the use of NVP-AUY922 in the early stage of clinical trial for NSCLC patients showed disappointing results (reviewed in [14,18,33,41,56]). Moreover, many such trials are associated with the occurrence of adverse effects instigated by administration of the inhibitory molecules (reviewed in [16,57,58]). Arguably, these effects may interfere with the anti-tumor activity of the blocking agents. In our present investigation, however, no discernible disturbances in the behavior and general condition of the tumor-bearing mice treated with all the tested inhibitors were observed during the daily inspections of the animals performed until the 7th day post completion of the treatments.

The third noticeable information begotten by the results of the present study is that concurrent application of WBI and any of the inhibitors did not seem to markedly affect the anti-neoplastic effect of the former treatment. Once again, the only exception to this general observation was the combined blockade of the functions of CTLA-4 and PD-1 immune checkpoints which, when added to the WBIs, exerted a notably more pronounced effect both in vivo (growth of the tumors in the lungs and under the skin) and in vitro (clonogenic potential of the tumor cells). Accordingly, although in terms of the intratumoral composition of immune cells and the serum levels of the tested cytokines, the addition of most of the inhibitors to WBI did not seem to generate any significant departures from the changes seen after the sole use of WBI, simultaneous blockade of the CTLA-4 and PD-1 immune checkpoints combined with WBI at both total doses of X-rays seemed to step up, if generally not significantly, the effects of the sole use of the latter treatments.

The lack of the significant synergistic or additive effect of the inhibitors added to the WBI clearly contrasts with the existing experimental and clinical evidence, indicating that the efficiency of cancer immunotherapy based on the blockade of CTLA-4 and/or PD-1 is enhanced when the blockade is combined with standard radiation therapy (reviewed in [19,29]). For instance, in a recent preclinical study using a mouse model of prostate cancer which responded poorly to immune checkpoint inhibition, the therapy became effective when application of the anti-PD-1 or anti-PD-L1 antibodies was coupled with irradiation of the tumor graft at high doses of X-rays [59]. Likewise, in C57BL/6 mice bearing o.t. (intracranially) implanted GL261 glioma cells, improved survival was demonstrated when the anti-PD-1 therapy was combined with local irradiation of the tumor, whereas either treatment alone was less effective [60]. Additionally, earlier studies utilizing murine models of breast cancer demonstrated that local radiotherapy effectively inhibited growth of the induced primary tumors, especially when the irradiations were associated with blockade of the function of CTLA-4. Importantly, in these studies, no anti-tumor effect was detected when the anti-CTLA-4 antibody was used as a sole treatment [61,62]. In the present investigation, in which we used a different model of the transplantable cancer and the ultra-low dose WBIs rather than high-dose localized radiation, addition of the blockers of CTLA-4, PD-1, and/or HSP90 hardly improved the well-pronounced anti-neoplastic effect of WBI. It is likely, therefore, that the low level irradiations did not evoke the activity of the ICs and/or HSP(s). Indeed, there are reports indicating that irradiation at 75 and 150 mGy of X-rays down-regulates the expression of CTLA-4 on splenocytes or Treg cells, respectively, obtained from the irradiated mice [63,64]. In our present study, we did not estimate the expression of the immune checkpoint or HSP molecules but we showed that multiple WBIs at 0.02 or 0.2 Gy of X-rays resulted in the reduction of the numbers of Treg and pro-inflammatory Th17 lymphocytes in the neoplastic tissues induced by implantation of LLC1 cells. This observation may contrast with the results of the standard high-dose radiotherapy which induces immunogenic death of cancer cells and inflammation and up-regulates Treg cells and/or the heat shock response (reviewed in [19,56,65]).

One limitation of our present study is the use of a single type of an ‘artificial’ neoplasm and a single strain of the mice used as recipients of the in vitro cultured Lewis lung cancer cells. Moreover, although the employed simple schedule of the administration of WBI relative to inhibition of the tested molecules produced reproducible and comparable results in mice transplanted with the tumor cells by three different routes, other time windows, dosing, frequency, and routes of applications, etc. should be tested in the context of the WBI coupled with immune checkpoint and/or heat shock protein blockade. These and other possible limitations of the study are amenable to be eliminated and should be addressed in future investigations.

In summary, the present results confirm and expand our previously reported observations of the anti-neoplastic activity of multiple whole-body irradiations of mice at low doses of X-rays by demonstrating that such WBIs at five daily doses of 0.02 and 0.2 Gy suppress the growth of Lewis lung cancer cells in vivo and in vitro and that the effects are accompanied by the relevant changes in the numbers of both pro- and anti-neoplastic immune cells in the developed tumors as well as in the levels of the selected pro- and anti-inflammatory cytokines in the serum of the irradiated mice. For the first time, however, the results indicate that a combination of WBIs with blockade of the immune checkpoints such as CTLA-4 and PD-1, especially when the two had been inactivated together, also potently inhibited the growth of lung cancer cells in vivo and their clonogenic potential in vitro. Such a combined treatment seemed to promote the above described cellular and cytokine alterations induced by the sole use of WBIs. Generally, inactivation of the function of heat shock protein HSP90 applied concurrently with WBI appeared to be less effective in these regards. Notably, the anti-neoplastic effects in vivo of WBIs used solely and in combination with the blocking agents were comparably expressed in mice with tumors induced by i.v., o.t., or s.c. injection of the LLC1 cells. Alas, the observed shifts in the composition of the immune cells and secreted cytokines addition of any or all of the tested blockers to WBI did not seem to markedly augment the already well pronounced anti-neoplastic effect of the latter. Moreover, application of most of the inhibitors without the concomitant WBI appeared to be almost totally ineffective in inhibiting the development of the transplanted tumors in vivo; only simultaneous inactivation of CTLA-4 and PD-1 suppressed the potential of LLC1 cells to produce neoplastic colonies in vitro and to grow into subcutaneous tumors in vivo. Obviously, further studies are needed to unravel the mechanisms of the apparent resistance of the experimental lung carcinoma to the potentially curative effects of the blockade of the function of immune checkpoints and/or HSP90 applied separately or together with WBI. Understanding of such mechanisms may be key to the design of effective strategies of treatment of lung and other cancers based on inactivation of such molecular targets in combination with low-dose radiotherapy.

## 4. Materials and Methods

### 4.1. List of Labels Used in Figures and Tables

The following labels are used in all the figures and tables presented:
•  0 Gy •  sham-exposed mice; •  0.1 Gy •  mice exposed to WBI at 0.1 Gy total dose X-rays; •  1.0 Gy •  mice exposed to WBI at 1.0 Gy total dose X-rays; •  PBS •  mice i.p. injected thrice a week with 5% glucose solution in PBS; •  anti-CTLA-4 •  mice i.p. injected thrice a week with molecules blocking the CTLA-4 receptor; •  anti-PD-1 •  mice i.p. injected thrice a week with molecules blocking the PD-1 receptor; •  NVP-AUY922•  mice i.p. injected thrice a week with molecules blocking the HSP90 protein;•  anti-CTLA-4 + NVP-AUY922 •  mice i.p. injected thrice a week with molecules blocking the CTLA-4 receptor and HSP90 protein; •  anti-PD-1 + NVP-AUY922 •  mice i.p. injected thrice a week with molecules blocking PD-1 and HSP90 protein; •  anti-CTLA-4 + anti-PD-1 •  mice i.p. injected thrice a week with molecules blocking CTLA-4 and PD-1 receptors; •  anti CTLA-4 + anti PD-1 + NVP-AUY922 •  mice i.p. injected thrice a week with molecules blocking CTLA-4 and PD-1 receptors, and HSP90 protein.•  TILs•  tumor infiltrating CD3+CD45+ lymphocytes•  TAMs•  tumor associated CD11b+Ly6C+Ly6G+ macrophages•  NK cells•  CD49b+CD335+ NK lymphocytes •  CTLs•  CD8+Cd178+ cytotoxic T lymphocytes•  Tregs•  CD4+CD25+FoxP3+ regulatory T lymphocytes •  Th17 cells•  CD4+IL17A+ helper T lymphocytes labelled as •  CD4+•  lymphocytes labelled with anti-CD4 monoclonal antibody•  CD8+•  lymphocytes labelled with anti-CD8 monoclonal antibody

### 4.2. Animals

**Figure 7:**
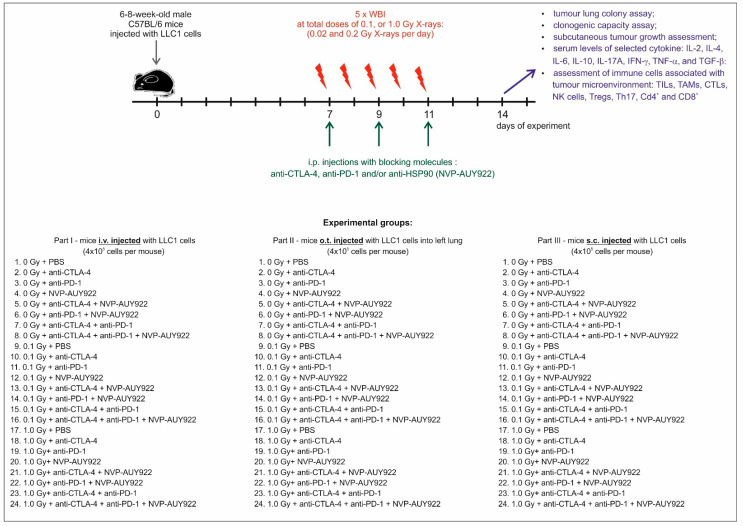
Experimental protocols.

Mice C57BL/6 were obtained from the Mossakowski Medical Research Institute of the Polish Academy of Sciences, Warsaw, Poland, and at 6–8 weeks of age were used for the experiments. The animals were housed under specific pathogen-free conditions in a Modular Animal Caging System^®^-MACS Mobile Units (Alternative Design, Siloam Springs, AR, USA), and were provided with a natural daily cycle (12-h photoperiod) and the ad libitum access to food and water. The living conditions and health of the animals were monitored daily by a veterinarian. The experiments were carried out so as to inflict the least pain on the animals. For all the procedures, the mice were anesthetized and/or sacrificed with isoflurane in the euthanasia induction box (WITKO, Lodz, Poland). All experimental procedures described below are outlined in Figure 1. The study was divided into three parts. In each part of the study, mice were randomly assigned to 24 experimental groups (see Figure 7) which were exposed to different doses of X-rays and administered, in different configurations, blockers of the immune checkpoints CTLA-4 and PD-1 and/or of HSP-90. Random numbers were generated using the standard = RAND() function. The total number of mice used in the present study equaled to 912. The investigations were carried out by permission of the 2nd Local Ethical Committee for Experimentation on Animals at the Warsaw University of Life Sciences in Warsaw, Poland (permission No. WAW2/065/2018 of 27 April 2018). All the experiments were performed in accordance with all the relevant guidelines and regulations. We also confirm that we complied with the ARRIVE guidelines.

### 4.3. Tumor Cells

Syngeneic Lewis Lung Carcinoma (LLC1) cells (ATCC; The Global Bioresource Center, LGC Standards, Kielpin Lomianki, Poland)—the only reproducible experimental model for the human non-small-cell lung cancer to date [46,66]—were used for the induction of tumor nodules in mice. The cells were maintained as a mono-layer culture in a culture medium composed of the DMEM W/GLUTAMAX-I, PYR, 4,5 GLU medium (GIBCO, LifeTechnologies, Warsaw, Poland), 10% Heat-innactivated FBS (PAN BIOTECH, IMMUNIQ, Zory, Poland), 100 U/mL penicillin, and 100 U/mL streptomycin (Corning, Sigma-Aldrich, Poznan, Poland) in standard conditions (SC): humidified atmosphere of 95% air and 5% CO2 at 37 °C. The LLC1 cells were passaged every two days using 0.25% Tripsin-EDTA (GIBCO). The cell line was tested free of mycoplasma.

### 4.4. Injection of LLC1 Cells

On day 0 of the experiment, after obtaining a single cell suspension using 0.25% Tripsin-EDTA, the LLC1 cells were counted in a mammalian cell counter NucleoCounter NC-100 (ChemoMetec, Allerød, Denmark) and brought to the required concentration. Then, three different ways of the injection of LLC1 cells into mice were used (see Figure 7):

1. intravenous (i.v.)—the animals were placed in a mouse holder and 4 × 10^5^ LLC1 cells per mouse suspended in 200 µL PBS (ADLAB, Lublin, Poland) were injected into a tail vain using a 1 mL insulin syringe (Becton Dickinson, Warsaw, Poland) with a hypodermic needle. The mice were then divided into 24 groups at 16 mice per group (total number of mice);
2. orthotopic (o.t.)—mice were anesthetized and put on their right flank. One-mL tuberculin syringes (Becton Dickinson) with hypodermic needles were used to inject 4 × 10^5^ cells per mouse in 100 µL PBS containing 1.35 mg/mL of Matrigel (VWR, Gdansk, Poland). The cells were injected percutaneously into the left lung at the lateral dorsal axillary line 1.5 cm above the lower rib line just below the inferior border of the scapula. The needle was quickly advanced 5–7 mm into the thorax and quickly removed after the injection of the cell suspension and the mouse was put back on the left flank. The treated animals were observed for 45–60 min until fully recovered. Finally, the mice were divided into 24 groups at 16 mice per group (total number of mice 384);
3. subcutaneous (s.c.)—4 × 10^5^ LLC1 cells per mouse in 200 µL PBS were injected directly into the lower back using the 1-mL tuberculin syringes (Becton Dickinson) with 30-gauge hypodermic needles. Mice were then divided into 24 groups at 6 mice per group (total number of mice 144).

### 4.5. Irradiation

Seven days after the injection of the LLC1 cell, the animals were exposed to fractionated WBI with X-rays at the total dose of 0.1 or 1.0 Gy given in five daily fractions (0.02 or 0.2 Gy per fraction per day)—see Figure 4. All the irradiations were performed at the Institute of Nuclear Chemistry and Technology, Warsaw, Poland, using the Xylon International Smart 200 X-ray generator (Xylon, San Jose, CA, USA) at 200 kV, 2 mA, 0.09 Gy/min dose rate. During the irradiations, the animals were placed in a ventilated container and positioned along the axis of the radiation beam. Control mice were sham-exposed (generator at the off-mode) in identical conditions. The absorbed doses were verified using thermoluminescent dosimeters as described previously [67].

### 4.6. Application of the Blocking Molecules

Molecules against the CTLA-4 (anti-mouse CTLA-4 antibody-clone 9H10; Hoelzel, Cologne, Germany) and PD-1 (anti-mouse PD-1 antibody-clone RMP1-14; Hoelzel) immune checkpoint receptors and/or the HSP90 protein (NVP-AUY922; MedChemTronica, Sollentuna, Sweden) were injected intraperitoneally (i.p.) thrice a week (on days 7, 9, and 11 after inoculation of LLC1 cells) during the period of time when the irradiations were performed (see Figure 4). The blocking molecules were dissolved in the 5% glucose solution (Sigma-Aldrich) in PBS and administered at the following concentrations: 10 mg/kg body mass of anti-mouse CTLA-4 or anti-mouse PD-1 antibodies and 15 mg/kg body mass of NVP-AUY922. The molecules were administered in different configurations (see Figure 4).

### 4.7. Lung Tumor Colony Assay

Fourteen days after the i.v., o.t., or s.c. inoculation of LLC cells, mice were sacrificed, their lungs were filled with the solution of India ink and formalin, and the number of visible macroscopic tumor colonies per lung were counted using a magnifying glass [67]. Each experimental group that was i.v. or o.t. injected with tumor cells consisted of ten mice, whereas each experimental group that was s.c. injected with tumor cells consisted of three mice.

In addition, potential metastases were macroscopically assessed using a magnifying glass in the spleens and livers which were collected from mice of the above groups.

### 4.8. Tissue Collection

Fourteen days after the i.v., o.t., or s.c. inoculation of LLC1 cells, mice were sacrificed, their lungs, spleens, livers, and subcutaneously growing tumors were removed, homogenized, and suspended in culture medium. Cell suspensions of the lung, spleen, and liver homogenates were divided in two parts. One part was used for clonogenic capacity assays, whereas the second part as well as cell suspensions of the subcutaneously growing tumors was used for flow cytometric analysis of the numbers of cells of the innate and adaptive immunity associated with cancer progression. The cells assayed for the clonogenic capacity assays were utilized immediately after collection, whereas those destined for analysis by flow cytometry were frozen at −80 °C.

Serum samples were prepared from peripheral blood obtained by heart punctures of the anaesthetized mice 14 days after the i.v., o.t., or s.c. inoculation of LLC1 cells. The obtained serum samples were frozen at −80 °C

Each experimental group of mice that was i.v. or o.t. injected with tumor cells consisted of six animals, whereas each experimental group of mice that was s.c. injected with tumor cells consisted of three animals.

### 4.9. Clonogenic Capacity

The cell suspensions obtained from the lungs, spleens, and livers of mice i.v., o.t., or s.c. inoculated with LLC1 cells were seeded on the 6-well culture plates (4 mL per well for 2 wells). The plates were inspected daily, the CM being replaced every three days. Fourteen to 21 days later, the medium was removed and the wells were rinsed with PBS. The developed tumor cell colonies (clones) were fixed and stained with a mixture of 0.5% crystal violet (Sigma-Aldrich) in 50/50 methanol (Avantor Performance Materials Poland, Gliwice, Poland)/water for 30 min. On the following day, the clones were counted and expressed as total number of neoplastic colonies per organ according to the formulas:

total no. of clones per lung = (no. of clones in well 1 + no. of clones in well 2) × 2(1)

total no. of clones per spleen = (no. of clones in well 1 + no. of clones in well 2) × 2(2)

total no. of clones per liver = (no. of clones in well 1 + no. of clones in well 2) × 3(3)

After estimation of the total number of the clones per organ, the tumor clonogenic capacity (CC) was calculated using the following equation:

CC = [(mean no. of clones per organ obtained from mice of the treated group)/(mean no. of clones per organ obtained from mice of the untreated group)] × 100%(4)

Each experimental group of mice which were i.v. or o.t. injected with tumor cells consisted of six animals, whereas each experimental group of mice which were s.c. injected with tumor cells consisted of three animals.

### 4.10. Tumor Volume Assessment

Mice s.c. inoculated with LLC1 cells were identified by the ear tags (VIVARI, Warsaw, Poland). The growing tumors were measured manually at regular intervals using the electronic calipers and the tumor volumes were estimated using the formula:

tumor volume = 4π/3 × w × h × l(5)

where width (w), height (h), and length (l) are the three largest diameters. Each experimental group consisted of six mice.

### 4.11. Flow Cytometric Analysis

To analyze the cells of the innate and adaptive immunity associated with cancer progression such as TAMs, TILs, Tregs, Th17 lymphocytes, CTLs, and NK cells, defrosted cells obtained from the lungs and subcutaneously growing tumors were assayed in the FACS Calibur flow cytometer (Becton Dickinson, Warsaw, Poland) using the following monoclonal antibodies: FITC Rat Anti-Mouse CD45, APC Rat Anti-CD11b, PE Rat Anti-Mouse Ly-6G and Ly-6C, PerCP-Cy™5.5 Hamster Anti-Mouse CD3e, PerCP-Cy™5.5 Rat Anti-Mouse CD8a, PE Hamster Anti-Mouse CD178, FITC Rat Anti-Mouse CD49b, Alexa Fluor^®^ 647 Rat Anti-Mouse CD335 (NKp46), and Mouse Th17/Treg Phenotyping Kit (all from BD Pharmingen), BB515 Rat Anti-Mouse CD25 (BD Horizon).

After defrosting, the obtained serum samples, after defrosting, were assayed for the levels of: IL-2, IL-4, IL-6, IL-10, IL-17A, IFN-γ, and TNF-α, using the Cytometric Bead Array (CBA) Mouse Th1/Th2/Th17 Cytokine Kit (BD).

All determinations were performed in triplicates. The obtained data were analyzed with the use of the CellQuest software (Becton-Dickinson) and FCAP Array software (Becton-Dickinson).

### 4.12. ELISA Tests

After defrosting, the obtained serum samples, after defrosting, were assayed for the levels of TGF-β using the Mouse TGF Beta 1 ELISA Kit PicoKineTM (Boster Bio, Pleasanton, CA, USA) with use of ELISA method employing the Epoch™ microplate spectrophotometer (BioTek Instruments, Inc., Winooski, VT, US) and the ELx50™ microplate strip washer (BioTek Instruments). All determinations were performed in triplicates. The obtained data were analyzed with the use of a reader software Gen5 2.0 (BioTek Instruments).

### 4.13. Statistical Analysis

Inter-group differences in the number of tumor colonies, tumor clonogenic capacities, tumor volumes, percentages of immune cells, and serum levels of cytokines were analyzed using the Mann –Whitney U test for non-parametric trials with *p* < 0.05 regarded as significant.

## 5. Conclusions

In the present study, we aimed at determining whether blockade of the activity of one or two immune checkpoints and/or HSP90 can modify the previously reported by us and other authors of the anti-cancer effect of whole-body irradiations at low doses of X-rays (WBIs). The obtained results demonstrate for the first time that such targeted blockades used in conjunction with WBI, especially when the blockers of the CTLA-4 and PD-1 immune checkpoints were inactivated together, also potently inhibited the growth of the Lewis lung cancer (LLC1) cells in vivo and their clonogenic potential in vitro. Accordingly, such a combined treatment led to the relevant alterations in the numbers of pro- and anti-neoplastic immune cells infiltrating the induced tumors as well as in the production of anti- and pro-inflammatory cytokines likely involved in the tumor progression. Notably, the observed anti-neoplastic effects of the combined treatments were comparably expressed in mice i.v., o.t., or s.c. injected with LLC1 cells. However, application of any one or any combination of the tested blockers without the parallel WBI was hardly effective, despite the accompanying shifts in the composition of immune cells and secreted cytokines. Clearly, further studies are needed to elucidate the impact of the tested therapeutic modalities on the neoplastic cells and other immunologically-relevant elements of the tumor microenvironment. The results of such studies, along with determination of the optimal dosage and schedule of the delivery of the tested modalities will be crucial for designing an effective anti-cancer strategy based on the combination of targeted treatments with low-dose radiotherapy.

## Figures and Tables

**Table 1:** Clonogenic capacity (CC) in vitro of tumor cells obtained from the lungs of C57BL/6 mice(intravenously) i.v. injected with LLC1 cells and treated with blockers of the immune checkpoints and/or heat shock protein 90 (HSP90) combined with whole-body irradiations (WBI) at total doses of 0.1 and 1.0 Gy. Results are expressed as percentages of the control value (100%).

Groups	0 Gy	0.1 Gy	1.0 Gy
PBS	100.0	65.1 *^	72.8 *^
anti-CTLA-4	80.4	50.6 *^	61.9 *^
anti-PD-1	90.2	60.9 *^	69.8 *^
NVP-AUY922	91.9	62.1 *^	69.4 *^
anti-CTLA-4 + NVP-AUY922	81.3	53.2 *^	62.1 *^
anti-PD-1 + NVP-AUY922	103.0	70.6 *^	79.6 ^
anti-CTLA-4 + anti-PD-1	67.2 *	43.4 *^	50.7 *^
anti-CTLA-4 + anti-PD-1 + NVP-AUY922	95.3	66.8 *^	81.3

The results obtained from experiments conducted on six mice per group are presented. * indicates statistically significant (*p* < 0.05) difference from the results obtained in the sham-exposed mice injected with PBS; ^ indicates statistically significant (*p* < 0.05) difference from the results obtained in the sham-exposed mice injected with the same molecule or combination of the molecules.

**Table 2:** Clonogenic capacity (CC) in vitro of tumor cells obtained from the lungs of C57BL/6 mice orthotopically (o.t.) injected with LLC1 cells and treated with blockers of the immune checkpoints and/or HSP90 combined with WBI at total doses of 0.1 and 1.0 Gy. Results are expressed as percentages of the control value (100%).

Groups	0 Gy	0.1 Gy	1.0 Gy
PBS	100.0	70.5 *^	81.1
anti-CTLA-4	92.1	56.8 *^	66.2 *^
anti-PD-1	98.6	64.0 *^	77.7 *
NVP-AUY922	90.6	61.9 *^	71.7 *
anti-CTLA-4 + NVP-AUY922	86.8	69.1 *^	74.1 *
anti-PD-1 + NVP-AUY922	103.6	70.5 *^	78.4
anti-CTLA-4 + anti-PD-1	63.5 *	47.2 *^#	54.0 *^^#^
anti-CTLA-4 + anti-PD-1 + NVP-AUY922	102.2	72.7 *^	85.6

The results obtained from experiments conducted on six mice per group are presented. All abbreviations are explained in Materials and Methods. * indicates statistically significant (*p* < 0.05) difference from the results obtained in the sham-exposed mice injected with PBS; ^ indicates statistically significant (*p* < 0.05) difference from the results obtained in the sham-exposed mice injected with the same molecule or combination of the molecules; # indicates statistically significant (*p* < 0.05) difference from the results obtained in mice injected with PBS and exposed to the respective WBI.

**Table 3:** The CD4+/CD8+ T lymphocyte ratios among the cells obtained from the lungs after i.v. or o.t. injection of LLC1 cells or from tumors grown subcutaneously in C57BL/6 mice treated with blockers of the immune checkpoints and/or HSP90 combined with WBI at total doses of 0.1 and 1.0 Gy.

Method of Injection of LLC1 Cells	Groups	CD4+/CD8+ Ratio		
		0 Gy	0.1 Gy	1.0 Gy
i.v.	PBS	5.9	4.4	4.4
	anti-CTLA-4	5.7	4.5	5.2
	anti-PD-1	5.8	5.2	5.1
	NVP-AUY922	5.8	5.3	5.1
	anti-CTLA-4 + NVP-AUY922	5.8	4.9	4.9
	anti-PD-1 + NVP-AUY922	5.9	5.0	5.1
	anti-CTLA-4 + anti-PD-1	5.6	3.5	3.8
	anti-CTLA-4 + anti-PD-1 + NVP-AUY922	5.7	5.1	5.2
o.t.	PBS	6.1	4.3	3.3
	anti-CTLA-4	6.2	4.8	3.3
	anti-PD-1	6.1	5.6	4.1
	NVP-AUY922	6.1	5.6	4.1
	anti-CTLA-4 + NVP-AUY922	6.1	5.3	3.8
	anti-PD-1 + NVP-AUY922	6.2	5.3	3.8
	anti-CTLA-4 + anti-PD-1	6.0	3.8	1.6
	anti-CTLA-4 + anti-PD-1 + NVP-AUY922	6.0	5.4	3.9
s.c.	PBS	5.4	4.5	3.3
	anti-CTLA-4	5.2	5.6	4.1
	anti-PD-1	5.1	5.4	3.9
	NVP-AUY922	5.1	5.4	3.8
	anti-CTLA-4 + NVP-AUY922	4.9	5.3	3.8
	anti-PD-1 + NVP-AUY922	5.1	5.4	3.9
	anti-CTLA-4 + anti-PD-1	3.8	3.6	1.6
	anti-CTLA-4 + anti-PD-1 + NVP-AUY922	5.2	5.3	3.8

The results obtained from experiments conducted on six (i.v. or o.t. injection of LLC1 cells) or three (s.c. injection) mice per group are presented. All abbreviations are explained in Materials and Methods.

## Data Availability

The data presented in this study are available on request from the authors.

## Supplementary Materials

The following are available online at https://www.mdpi.com/article/10.3390/ijms22126309/s1.
