# Supplementary material for: Effects of a Unique Combination of the Whole-Body Low Dose Radiotherapy with Inactivation of Two Immune Checkpoints and/or a Heat Shock Protein on the Transplantable Lung Cancer in Mice
Source: Int J Mol Sci. 2021 Jun 11;22(12):6309. doi: 10.3390/ijms22126309 (PMC8231142; doi:10.3390/ijms22126309)
Supplement: Supplementary file 1 [file ijms-22-06309-s001.zip › ijms-1252950-supplementary.pdf]

Supplementary Materials for:

Effects of a Unique Combination of the Whole-Body Low Dose Radiotherapy With Inactivation of Two Immune Checkpoints and/or a Heat Shock Protein on the Transplantable Lung Cancer in Mice

Ewa M. Nowosielska [1,†,*], Aneta Cheda [1,†], Mateusz Pociegiel [2], Lukasz Cheda [3], Pawel Szymanski [1,4], and Antoni Wiedlocha [1,5,6]

[1] Department of Radiobiology and Radiation Protection, Military Institute of Hygiene and Epidemiology, 4 Kozielska St., 01-163 Warsaw, Poland; aneta.cheda@wihe.pl (A.C.); pawel.szymanski@wihe.pl (P.S.);

[2] National Centre for Nuclear Research Radioisotope Centre POLATOM, 7A. Soltana St., 05-400 Otwock, Poland; mateusz.pociegiel@ncbj.gov.pl

[3] Faculty of Chemistry, Biological and Chemical Research Centre, University of Warsaw, 101 Żwirki i Wigury St., 02-089 Warsaw, Poland; lcheda@chem.uw.edu.pl

[4] Department of Pharmaceutical Chemistry, Drug Analyses and Radiopharmacy, Faculty of Pharmacy, Medical University of Lodz, 1 Muszyńskiego St., 90-151 Lodz, Poland

[5] Department of Molecular Cell Biology, Institute for Cancer Research, The Norwegian Radium Hospital, Oslo University Hospital, Montebello, 0379 Oslo, Norway; Antoni.wiedlocha@ous-research.no

[6] Centre for Cancer Reprograming, Institute of Clinical Medicine, Faculty of Medicine, University of Oslo, Montebello, 0379 Oslo, Norway

[†] These authors contributed equally to this work

* Correspondence: ewa.nowosielska@wihe.pl; Tel.: +48 261853066

Table S1. Serum level of TGF-β in C57BL/6 mice after i. v., o.t., or s.c. injection of LLC1 and treatment with blockers of the immune checkpoints and/or HSP90 combined with WBI at total doses of 0.1 or 1.0 Gy.

Method of injection of LLC1 cells	Groups	TGF- β [pg/ml]		
		**0 Gy**	**0.1 Gy**	**1.0 Gy**
i.v.	PBS	182.5	63.2*	74.5*
	anti-CTLA-4	177.5	92.3*^	89.8*^
	anti-PD-1	178.6	90.3*^	88.8*^
	NVP-AUY922	182.6	124.8*^	100.2*^
	anti-CTLA-4 + NVP-AUY922	170.6	112.0*^	103.7*^
	anti-PD-1 + NVP-AUY922	178.2	96.9*^	92.3*^
	anti-CTLA-4 + anti-PD-1	171.2	57.4*^	50.3*^
	anti-CTLA-4 + anti-PD-1 + NVP-AUY922	172.6	118.8*^	114.6*^
o.t.	PBS	173.1	46.8*	57.0*
	anti-CTLA-4	164.2	77.1*^	74.2*^
	anti-PD-1	158.6	75.1*^	71.9*^
	NVP-AUY922	156.8	109.1*^	84.6*^
	anti-CTLA-4 + NVP-AUY922	154.3	91.4*^	87.3*^
	anti-PD-1 + NVP-AUY922	164.8	78.0*^	77.1*^
	anti-CTLA-4 + anti-PD-1	162.0	39.6*^	33.3*^
	anti-CTLA-4 + anti-PD-1 + NVP-AUY922	175.9	103.1*^	99.0*^
s.c.	PBS	159.7	37.8*	57.0*
	anti-CTLA-4	155.8	68.3*^	74.2*^
	anti-PD-1	150.0	66.9*^	71.9*^
	NVP-AUY922	148.6	100.4*^	84.6*^
	anti-CTLA-4 + NVP-AUY922	145.8	82.1*^	87.3*^
	anti-PD-1 + NVP-AUY922	156.1	69.8*^	77.1*^
	anti-CTLA-4 + anti-PD-1	143.5	52.0*^	33.3*^
	anti-CTLA-4 + anti-PD-1 + NVP-AUY922	146.7	94.7*^	99.0*^

Mean values obtained from experiments conducted on six (i.v. or o.t. injection of tumour cells) or three (s.c. injection of tumour cells) mice per group are presented. All abbreviations are explained in 'Materials and Methods.' *indicates statistically significant ($p<0.05$) difference from the results obtained in the sham-exposed mice injected with PBS; ^indicates statistically significant ($p<0.05$) difference from the results obtained in the sham-exposed mice injected with the same molecule or combination of the molecules.

Table S2. Serum levels of IL-10 in C57BL/6 mice after i.v., o.t., or s.c. injection of LLC1 and treatment with blockers of the immune checkpoints and/or HSP90 combined with WBI at total doses of 0.1 and 1.0 Gy.

Method of injection of LLC1 cells	Groups	IL-10 [pg/ml]		
		0 Gy	0.1 Gy	1.0 Gy
i.v.	PBS	16.37	7.83*	7.50*
	anti-CTLA-4	14.11	9.82*^	9.14*^
	anti-PD-1	13.83	10.16*^	9.48*^
	NVP-AUY922	13.65	11.55*^	10.86*^
	anti-CTLA-4 + NVP-AUY922	13.41	9.14*^	9.14*^
	anti-PD-1 + NVP-AUY922	14.99	10.51*^	10.86*^
	anti-CTLA-4 + anti-PD-1	14.17	7.37*^	7.16*^
	anti-CTLA-4 + anti-PD-1 + NVP-AUY922	16.50	8.14*^	9.48*^
o.t.	PBS	8.34	4.61*	4.28*
	anti-CTLA-4	7.65	5.27*^	5.93*^
	anti-PD-1	7.65	5.27*^	4.93*^
	NVP-AUY922	7.30	5.27*^	5.60*^
	anti-CTLA-4 + NVP-AUY922	6.95	4.73*^	4.60*^
	anti-PD-1 + NVP-AUY922	7.61	5.93*^	4.16*^
	anti-CTLA-4 + anti-PD-1	7.27	3.62*^	3.29*^
	anti-CTLA-4 + anti-PD-1 + NVP-AUY922	6.89	5.93*^	3.95*^
s.c.	PBS	5.93	3.29*	2.96*
	anti-CTLA-4	5.83	4.73*^	3.87*^
	anti-PD-1	5.43	4.28*^	3.73*^
	NVP-AUY922	5.93	4.61*^	3.53*^
	anti-CTLA-4 + NVP-AUY922	5.60	4.16*^	3.81*^
	anti-PD-1 + NVP-AUY922	5.74	3.95*^	3.20*^
	anti-CTLA-4 + anti-PD-1	5.27	2.90*^	2.33*^
	anti-CTLA-4 + anti-PD-1 + NVP-AUY922	5.57	3.87*^	3.53*^

Mean values obtained from experiments conducted on six (i.v. or o.t. injection of tumour cells) or three (s.c. injection of tumour cells) mice per group are presented. All abbreviations are explained in 'Materials and Methods.' *indicates statistically significant ($p<0.05$) difference from the results obtained in the sham-exposed mice injected with PBS; ^indicates statistically significant ($p<0.05$) difference from the results obtained in the sham-exposed mice injected with the same molecule or combination of the molecules.

Table S3. Serum levels of TNF-α in C57BL/6 mice after i.v., o.t., or s.c. injection of LLC1 and treatment with blockers of the immune checkpoints and/or HSP90 combined with WBI at total doses of 0.1 and 1.0 Gy.

**Method of injection of LLC1 cells**	**Groups**	**TNF-α [pg/ml]**		
		**0 Gy**	**0.1 Gy**	**1.0 Gy**
i.v.	PBS	11.44	13.37^{*}	13.31^{*}
	anti-CTLA-4	11.44	$12.68^{*\wedge}$	$12.67^{*\wedge}$
	anti-PD-1	10.86	$12.67^{*\wedge}$	$12.23^{*\wedge}$
	NVP-AUY922	11.24	$12.45^{*\wedge}$	$12.04^{*\wedge}$
	anti-CTLA-4 + NVP-AUY922	11.05	$12.68^{*\wedge}$	$11.84^{\wedge}$
	anti-PD-1 + NVP-AUY922	11.05	$11.64^{\wedge}$	$12.68^{*\wedge}$
	anti-CTLA-4 + anti-PD-1	10.86	$13.61^{*\wedge}$	$13.75^{*\wedge}$
	anti-CTLA-4 + anti-PD-1 + NVP-AUY922	11.24	$12.88^{*\wedge}$	$13.31^{*\wedge}$
o.t.	PBS	13.85	38.48^{*}	37.76^{*}
	anti-CTLA-4	15.86	$35.46^{*\wedge}$	$28.07^{*\wedge}$
	anti-PD-1	14.09	$19.85^{*\wedge}$	$16.97^{*\wedge}$
	NVP-AUY922	15.13	$19.28^{*\wedge}$	$17.83^{*\wedge}$
	anti-CTLA-4 + NVP-AUY922	15.61	$29.06^{*\wedge}$	$17.83^{*\wedge}$
	anti-PD-1 + NVP-AUY922	15.13	$23.46^{*\wedge}$	16.60^{*}
	anti-CTLA-4 + anti-PD-1	15.86	$59.67^{*\wedge}$	$57.62^{*\wedge}$
	anti-CTLA-4 + anti-PD-1 + NVP-AUY922	14.09	$25.85^{*\wedge}$	$35.46^{*\wedge}$
s.c.	PBS	8.05	10.13^{*}	10.29^{*}
	anti-CTLA-4	8.29	$9.93^{*\wedge}$	$10.11^{*\wedge}$
	anti-PD-1	8.11	$9.75^{*\wedge}$	$9.94^{*\wedge}$
	NVP-AUY922	8.05	$9.27^{*\wedge}$	$9.75^{*\wedge}$
	anti-CTLA-4 + NVP-AUY922	8.05	$9.18^{*\wedge}$	$9.39^{*\wedge}$
	anti-PD-1 + NVP-AUY922	7.94	$8.64^{*\wedge}$	$8.71^{*\wedge}$
	anti-CTLA-4 + anti-PD-1	8.54	$10.53^{*\wedge}$	$10.67^{*\wedge}$
	anti-CTLA-4 + anti-PD-1 + NVP-AUY922	8.37	$9.57^{*\wedge}$	$9.05^{*\wedge}$

Mean values obtained from experiments conducted on six (i.v. or o.t. injection of tumour cells) or three (s.c. injection of tumour cells) mice per group are presented. All abbreviations are explained in 'Materials and Methods.' *indicates statistically significant ($p<0.05$) difference from the results obtained in the sham-exposed mice injected with PBS; ^indicates statistically significant ($p<0.05$) difference from the results obtained in the sham-exposed mice injected with the same molecule or combination of the molecules.

Table S4. Serum levels of IFN-γ in C57BL/6 mice after i.v., o.t., or s.c. injection of LLC1 and treatment with blockers of the immune checkpoints and/or HSP90 combined with WBI at total doses of 0.1 and 1.0 Gy.

Method of injection of LLC1 cells	Groups	IFN – γ [pg/ml]		
		**0 Gy**	**0.1 Gy**	**1.0 Gy**
i.v.	PBS	1.83	5.95*	5.55*
	anti-CTLA-4	1.83	3.85*^	2.52*^
	anti-PD-1	1.77	2.58*^	3.01*^
	NVP-AUY922	1.72	2.93*^	2.39*^
	anti-CTLA-4 + NVP-AUY922	1.72	2.45*^	2.66*^
	anti-PD-1 + NVP-AUY922	1.71	2.13*^	2.14*^
	anti-CTLA-4 + anti-PD-1	1.60	5.26*^	5.37*^
	anti-CTLA-4 + anti-PD-1 + NVP-AUY922	1.60	3.21*^	2.90*^
o.t.	PBS	0.79	1.72*	1.72*
	anti-CTLA-4	0.89	1.47*^	1.39*^
	anti-PD-1	0.84	1.28*^	1.35*^
	NVP-AUY922	0.84	1.23*^	1.35*^
	anti-CTLA-4 + NVP-AUY922	0.89	1.71*^	1.33*^
	anti-PD-1 + NVP-AUY922	0.89	1.60*^	1.28*^
	anti-CTLA-4 + anti-PD-1	0.89	1.83*^	1.77*^
	anti-CTLA-4 + anti-PD-1 + NVP-AUY922	0.85	1.55*^	1.24*^
s.c.	PBS	0.61	1.08*	1.03*
	anti-CTLA-4	0.75	1.03*^	0.96*^
	anti-PD-1	0.70	0.89*^	0.90*^
	NVP-AUY922	0.66	0.93*^	0.90*^
	anti-CTLA-4 + NVP-AUY922	0.74	0.93*^	0.93*^
	anti-PD-1 + NVP-AUY922	0.70	0.89*^	0.93*^
	anti-CTLA-4 + anti-PD-1	0.79	1.23*^	1.23*^
	anti-CTLA-4 + anti-PD-1 + NVP-AUY922	0.84	0.85	0.89

Mean values obtained from experiments conducted on six (i.v. or o.t. injection of tumour cells) or three (s.c. injection of tumour cells) mice per group are presented. All abbreviations are explained in 'Materials and Methods.' *indicates statistically significant ($p<0.05$) difference from the results obtained in the sham-exposed mice injected with PBS; ^indicates statistically significant ($p<0.05$) difference from the results obtained in the sham-exposed mice injected with the same molecule or combination of the molecules.

Table S5. Serum levels of IL-6 in C57BL/6 mice after i.v., o.t., or s.c. injection of LLC1 and treatment with blockers of the immune checkpoints and/or HSP90 combined with WBI at total doses of 0.1 and 1.0 Gy.

Method of injection of LLC1 cells	Groups	IL-6 [pg/ml]		
		0 Gy	0.1 Gy	1.0 Gy
i.v.	PBS	8.94	8.79	9.11
	anti-CTLA-4	8.93	8.79	9.11
	anti-PD-1	8.57	8.79	9.08
	NVP-AUY922	8.39	8.61	9.08
	anti-CTLA-4 + NVP-AUY922	8.37	8.55	8.94
	anti-PD-1 + NVP-AUY922	8.18	8.44	8.62
	anti-CTLA-4 + anti-PD-1	8.64	8.27	8.62
	anti-CTLA-4 + anti-PD-1 + NVP-AUY922	8.37	8.27	8.12
o.t.	PBS	4.31	4.67	4.57
	anti-CTLA-4	4.43	4.37	4.63
	anti-PD-1	4.56	4.24	4.43
	NVP-AUY922	4.81	4.63	4.81
	anti-CTLA-4 + NVP-AUY922	4.31	4.71	4.57
	anti-PD-1 + NVP-AUY922	4.35	4.81	4.81
	anti-CTLA-4 + anti-PD-1	4.49	4.95	4.63
	anti-CTLA-4 + anti-PD-1 + NVP-AUY922	5.57	4.35	4.29
s.c.	PBS	0.65	0.59	0.42
	anti-CTLA-4	0.56	0.61	0.53
	anti-PD-1	0.47	0.52	0.43
	NVP-AUY922	0.56	0.50	0.33
	anti-CTLA-4 + NVP-AUY922	0.62	0.57	0.44
	anti-PD-1 + NVP-AUY922	0.52	0.50	0.54
	anti-CTLA-4 + anti-PD-1	0.59	0.54	0.36
	anti-CTLA-4 + anti-PD-1 + NVP-AUY922	0.52	0.42	0.46

Mean values obtained from experiments conducted on six (i.v. or o.t. injection of tumour cells) or three (s.c. injection of tumour cells) mice per group are presented. All abbreviations are explained in 'Materials and Methods.' *indicates statistically significant ($p<0.05$) difference from the results obtained in the sham-exposed mice injected with PBS; ^indicates statistically significant ($p<0.05$) difference from the results obtained in the sham-exposed mice injected with the same molecule or combination of the molecules.
